# Desarrollo de una política pública integral de prevención del embarazo en adolescentes en Uruguay

**DOI:** 10.26633/RPSP.2021.93

**Published:** 2021-08-12

**Authors:** Alejandra López-Gómez, Silvia Graña, Valeria Ramos, Leticia Benedet

**Affiliations:** 1 Universidad de la República Montevideo Uruguay Universidad de la República, Montevideo, Uruguay.; 2 Administración de Servicios de Salud del Estado Montevideo Uruguay Administración de Servicios de Salud del Estado, Montevideo, Uruguay.; 3 Salud Sexual y Reproductiva, Fondo de Población de las Naciones Unidas Montevideo Uruguay Salud Sexual y Reproductiva, Fondo de Población de las Naciones Unidas, Montevideo, Uruguay.; 4 Área de Género, Programa EUROsociAL+ Montevideo Uruguay Área de Género, Programa EUROsociAL+, Montevideo, Uruguay.

**Keywords:** Embarazo en adolescencia, política pública, Uruguay, Pregnancy in adolescence, public policy, Uruguay, Gravidez na adolescência, política pública, Uruguai

## Abstract

Se presentan las principales características y logros de la Estrategia Nacional e Intersectorial para la Prevención del Embarazo en Adolescentes, implementada en Uruguay entre 2016 y 2020. Este proceso se desarrolló en un contexto en el que el embarazo no intencional en adolescentes continúa siendo un problema social relevante para Uruguay y la Región, por lo que se requieren políticas públicas integrales, sostenidas y basadas en evidencias científicas. En Uruguay, la fecundidad adolescente se ha mantenido en niveles elevados por más de una década. Además de la acción intersectorial del Gobierno y la sociedad civil, la estrategia aprobada contó con asesoramiento científico desde el ámbito académico y la cooperación técnica y financiera de organismos regionales e internacionales. Las acciones y medidas adoptadas se basan en una visión socio-ecológica, con sensibilidad cultural, enfoque transformador de género y perspectiva de derechos humanos. Entre las barreras más importantes están las normas sociales que valoran la maternidad como el principal proyecto de vida para las mujeres que viven en contextos de pobreza, los estereotipos de género —el embarazo como una responsabilidad exclusiva de las adolescentes, sin involucrar a los adolescentes varones—, el estigma del aborto, la insuficiente oferta de servicios de salud sexual y reproductiva, y la resistencia a visibilizar el embarazo en niñas menores de 15 años víctimas de la violencia estructural e intrafamiliar. Es necesario asegurar la continuidad de las políticas públicas, ajustadas a un enfoque de género y de derechos humanos, y que se tomen en cuenta los nuevos escenarios, como el que impone la pandemia por COVID-19.

## Abstract (en)

This article presents the main characteristics and achievements of Uruguay’s National and Intersectoral Strategy for Prevention of Adolescent Pregnancy, implemented from 2016 to 2020. This strategy was implemented in a context in which unintentional adolescent pregnancy continues to be a major social problem for Uruguay and the Region, necessitating comprehensive, sustained, and evidence-based public policies. In Uruguay, adolescent fertility rates have remained high for over a decade. In addition to intersectoral action by government and civil society, the strategy received scientific assistance from the academic community, and technical and financial cooperation from regional and international organizations. Its actions and measures were adopted based on a socio-ecological vision, with cultural sensitivity, a gender-transformative approach, and a human rights perspective. Major barriers include social norms that value maternity as the main life project for women living in poverty, gender stereotypes (pregnancy as the exclusive responsibility of adolescent girls, without involving adolescent boys), the stigma of abortion, a lack of sexual and reproductive health services, and resistance to raising the visibility of pregnancy in girls under 15 years of age who are victims of structural and family violence. It is necessary to ensure the continuity of public policies––adjusted to a gender and human-rights approach––that take into account new scenarios such as the one imposed by the COVID-19 pandemic.

## RESUMO

Este artigo apresenta os principais aspectos e os resultados da *Estratégia Nacional e Intersetorial para Prevenção da Gravidez na Adolescência*, implementada no Uruguai entre 2016 e 2020. A gravidez não intencional na adolescência persiste como uma questão social importante no Uruguai e na Região, exigindo políticas públicas com base em evidências científicas que sejam abrangentes e permanentes. A fecundidade na adolescência no Uruguai tem se mantido elevada há mais de uma década. Além da ação intersetorial do governo e da sociedade civil, a estratégia aprovada recebeu orientação científica de entidades acadêmicas, e cooperação técnica e financeira de organismos regionais e internacionais. As ações e as medidas da estratégia foram elaboradas a partir de uma visão socioecológica, com sensibilidade cultural e enfoque transformador de gênero e uma perspectiva de direitos humanos. Entre as barreiras mais importantes enfrentadas estão as normas sociais que valorizam a maternidade como principal projeto de vida para a mulher que vive em situação de pobreza, os estereótipos de gênero (a gravidez é vista como responsabilidade exclusiva da adolescente, sem envolver o parceiro adolescente), o estigma do aborto, a oferta insuficiente de serviços de saúde sexual e reprodutiva, e a resistência a dar visibilidade à gravidez de menores de 15 anos que são vítimas de violência estrutural e intrafamiliar. É necessário garantir a continuidade das políticas públicas que incorporem uma perspectiva de gênero e direitos humanos, e que sejam adaptadas aos novos cenários da pandemia de COVID-19.

Varios gobiernos de América Latina han implementado estrategias para la reducción y prevención del embarazo no intencional en adolescentes (1), etapa de la vida que convencionalmente abarca a las personas de entre 10 y 19 años. Uruguay entró a formar parte de este grupo con la puesta en marcha en 2016 de la Estrategia Nacional e Intersectorial de Prevención del Embarazo No Intencional en Adolescentes, impulsada por el Ministerio de Salud Pública en articulación con otros organismos del Estado. La iniciativa contó con asesoramiento científico desde el ámbito académico y la cooperación técnica y financiera de organismos regionales e internacionales, como el Fondo de Población de las Naciones Unidas, la Organización Panamericana de la Salud, la Organización Mundial de la Salud, el Banco Interamericano de Desarrollo, el Fondo de las Naciones Unidas para la Infancia y el Programa para la Cohesión Social EUROsociAL+ (2). El diálogo fecundo entre los tomadores de decisión, la sociedad civil, el ámbito académico y la cooperación internacional fue uno de los factores clave para su elaboración y desarrollo (3).

Con esta política se buscó dar una respuesta integral e intersectorial a la aún alta tasa de fecundidad adolescente y su relación con las desigualdades sociales (4). El embarazo en adolescentes es un fenómeno multidimensional que requiere de análisis desde un modelo socio-ecológico (5), que permita distinguir los diversos niveles implicados y sus interrelaciones. La política adoptada se diseñó —desde este enfoque— para atender las diversas aristas de este complejo problema, sus determinantes y sus consecuencias, a fin de contribuir al bienestar de la población adolescente, promover sus derechos y acceso a los servicios de salud sexual y reproductiva, favorecer la educación sexual y fomentar entornos libres de discriminación y violencia de género. Para ello, su radio de acción se extiende a los adolescentes varones, las familias y las comunidades.

## RECONOCIMIENTO DE UN PROBLEMA SOCIAL: EMBARAZO EN ADOLESCENTES Y DESIGUALDADES SOCIALES

La adolescencia es una etapa crucial en el curso de la vida y para la sociedad constituye una segunda ventana de oportunidad para invertir en su desarrollo (6, 7). Internacionalmente, se reconoce que la salud sexual y reproductiva y la educación sexual de la población adolescente constituyen elementos centrales de ese desarrollo (8, 9). Las decisiones sexuales y reproductivas en la adolescencia tienen consecuencias directas para la salud, pueden comprometer oportunidades académicas, sociales y laborales inmediatas, y están influenciadas por la distribución y el acceso a los recursos económicos, educativos y sociales. En este sentido, el embarazo en adolescentes es también un problema social por ser expresión de las desigualdades estructurales, en particular de las desigualdades socioeconómicas, de género y étnico-raciales (10, 11).

En el período 2010-2015, la tasa estimada de fecundidad adolescente en América Latina y El Caribe era de 66,4 nacimientos por 1 000 adolescentes (de 15 a 19 años), muy por encima de esa tasa en Asia (42 por 1 000), América del Norte (27 por 1 000) y Europa (18 por 1 000) (12, 13).

A pesar de encontrarse en una posición relativamente ventajosa respecto a la Región, en 2014 Uruguay tenía una elevada tasa de fecundidad adolescente de 59 por 1 000 (3), la cual no había logrado reducir de manera significativa en lustros. Si bien ese año se constató un descenso en la tasa global de fecundidad a 1,9 nacimientos por mujer, los valores de la tasa en adolescentes eran similares a los registrados en 1996, cuando la tasa global era más elevada. Por ello, el peso relativo de la fecundidad adolescente en la tasa global aumentó en esos años (3).

En 2014, en algunas regiones del país se observaron indicadores similares a los de los países más pobres, lo que reflejaba la profunda segmentación socioeconómica y territorial existente en Uruguay. La maternidad en adolescentes era más frecuente en los sectores económicamente más vulnerables o con mayores necesidades básicas insatisfechas, situación agravada por el hecho de que tres de cada cuatro adolescentes madres habían abandonado sus estudios antes de su embarazo. La mayoría de las jóvenes de 25 a 29 años que fueron madres durante la adolescencia tenían menos de 9 años de estudios y se constató que la maternidad en las adolescentes no se veía acompañada por otros eventos de la transición a la vida adulta, como el empleo o la independencia del hogar de origen (3).

Según datos del Sistema Informático Perinatal de Uruguay correspondientes a los años 2013-2014, dos tercios de las adolescentes embarazadas entre 15 y 19 años declararon que el embarazo no fue planeado. Este porcentaje aumentaba después del parto: 71% de las adolescentes madres de 15 a 24 años declaró que hubiera preferido postergar la maternidad (3). Estos resultados hicieron cuestionar la idea predominante sobre la deseabilidad del embarazo en este sector de la población (12).

Para el año 2019, la tasa de fecundidad adolescente en Uruguay había descendido a 32 nacimientos por 1 000 adolescentes, en el marco de un acelerado descenso de la tasa global de fecundidad en el país. Aunque se han identificado algunos factores que podrían explicar este acelerado descenso en cinco años, se requieren más estudios para determinar el peso real de cada uno de ellos (2,14).

Otro fenómeno de interés evidenciado recientemente en la Región es el embarazo y la maternidad en niñas y adolescentes menores de 15 años. Si bien el número de nacimientos en este grupo de edad es menor que en el grupo de 15 a 19 años, su impacto resulta crítico dadas las graves consecuencias para la salud y el curso de la vida. Por otra parte, esta situación es producto de una cadena de vulneraciones de derechos y de la violencia estructural —en particular la violencia de género y sexual— que afecta a miles de niñas en el continente (15, 16). Entre los años 2015 y 2018, la tasa de fecundidad temprana (10-14 años) mostró una tendencia al descenso (de 1,0 a 0,6 nacimientos por 1 000 niñas/adolescentes de 10 a 14 años) con un leve aumento en 2019 (2).

El aborto voluntario se hizo legal en el país en noviembre del 2012 mediante la Ley 18.987, y los servicios de interrupción voluntaria del embarazo se iniciaron en el sistema nacional integrado de salud en enero del 2013. Los datos disponibles muestran que, del total de estos procedimientos, la proporción correspondiente a adolescentes de 15 a 19 años se mantuvo entre el 13% (2013) y el 17% (2019); en menores de 15 años este porcentaje estuvo entre 1,03% y 0,34% en ese mismo período (2). Estas cifras se deben ver en un contexto en el que se ha demostrado la existencia de barreras al acceso a estos servicios, relacionadas con el estigma del aborto y el temor a la sanción moral (17).

En el presente artículo se presentan las principales características y los logros de la política pública implementada entre los años 2016 y 2020 en Uruguay para prevenir y reducir el embarazo no intencional en adolescentes, así como los obstáculos para su implementación.

## DISEÑO Y PUESTA EN MARCHA DE LA ESTRATEGIA NACIONAL

En las últimas décadas, el Estado uruguayo contrajo varios compromisos internacionales relacionados con los derechos humanos, la igualdad de género, la salud y la educación, y en consecuencia se promulgaron leyes que establecen un marco normativo robusto para el desarrollo de una política pública dirigida a prevenir el embarazo no intencional en adolescentes (2, 18).

A partir de ese marco normativo y el diagnóstico de la situación, en 2016 se lanzó la correspondiente estrategia nacional, como política pública intersectorial, estructurada en cuatro componentes con objetivos a corto y mediano plazos.

- Componente 1, dirigido a la población adolescente en general. Objetivo: fortalecer las condiciones para el ejercicio de los derechos sexuales y reproductivos y la toma de decisiones con autonomía de las y los adolescentes, mediante la promoción de proyectos de vida diversos que cuestionen los modelos tradicionales de género.
- Componente 2, dirigido a adolescentes embarazadas. Objetivo: fortalecer los mecanismos de detección y captación temprana del embarazo en adolescentes para brindar la atención oportuna, ya sea para continuar o para interrumpir voluntariamente el embarazo, con el involucramiento de su entorno social cercano.
- Componente 3, dirigido a madres y padres adolescentes. Objetivo: garantizar el acceso a oportunidades, el ejercicio de sus derechos y la protección de las adolescentes embarazadas, madres y padres, con vistas a prevenir la ocurrencia de embarazos no intencionales reiterados.
- Componente 4, dirigido a la implementación intersectorial de la política pública. Objetivo: implementar de forma articulada a nivel nacional y local las políticas públicas vinculadas a la estrategia nacional aprobada (2).

Para su puesta en marcha se instaló una mesa nacional de coordinación intersectorial con los organismos involucrados, además de mesas locales en los territorios, una mesa de sistemas de información y una comisión de comunicación para conducir las campañas de información a la población. Entre otras medidas, se implementaron acciones de prevención adaptadas a las características culturales de los territorios; se capacitó a los equipos de salud y operadores territoriales; se realizaron campañas comunicacionales con la participación de adolescentes; se llevaron a cabo consultas con la sociedad civil organizada; se elaboraron directrices técnicas para la atención integral de adolescentes embarazadas menores de 15 años, incluida la atención psicoemocional; y se elaboró el flujograma para la atención a este sector de la población (2). Estas acciones reforzaron otras ya instrumentadas por los organismos participantes tendientes a promover la igualdad de género y el acceso a sus derechos, la disponibilidad de una amplia canasta de métodos anticonceptivos (incluidos los implantes subdérmicos) y servicios de salud sexual y reproductiva, la educación sexual en los distintos niveles de la educación pública y la protección social a las adolescentes madres. Todo esto implicó la planificación de actividades ajustadas a las necesidades y los requerimientos locales.

Los actores participantes en el diseño y la implementación de las acciones de la estrategia valoraron positivamente el desarrollo de una política pública con estas características. La estrategia nacional se nutrió del aporte de experiencias de países de la Región y Europa, que sirvieron de fuente de inspiración de buenas prácticas, al mismo tiempo que la experiencia de Uruguay ha resultado de interés para otros países que impulsan iniciativas similares (1, 2, 4).

Entre las buenas prácticas desarrolladas por la estrategia uruguaya se destacan la instalación del tema en la agenda pública, y el diseño de acciones culturalmente sensibles con la participación de la población adolescente, actores locales, profesionales de la salud y operadores territoriales, además de instituciones gubernamentales, sociales y académicas.

Como lección aprendida, se constató la existencia de barreras que obstaculizan la correcta implementación de lo aprobado, principalmente: a) la persistencia de normas sociales que valoran la maternidad como el principal proyecto de vida para las mujeres que viven en contextos de pobreza (3); b) los estereotipos de género que consideran el embarazo como una responsabilidad exclusiva de las adolescentes, sin involucrar a los adolescentes varones en las acciones de prevención; c) el estigma del aborto, que dificulta la toma de decisiones oportunas y el acceso a los servicios de interrupción voluntaria del embarazo; d) la insuficiente oferta de servicios de salud sexual y reproductiva, y su falta de adecuación a las necesidades de la población adolescente; y e) la resistencia a visibilizar el embarazo en niñas menores de 15 años víctimas de la violencia estructural de género y del abuso sexual intrafamiliar.

No obstante los logros alcanzados, es necesario avanzar en el diseño de intervenciones innovadoras basadas en evidencias científicas que permitan superar las barreras identificadas y que promuevan, en la población adolescente, las condiciones para el ejercicio de una sexualidad saludable y protegida. Para ello, es necesario examinar con mayor profundidad los factores sociales, cognitivos y emocionales que influyen en el comportamiento sexual y reproductivo de los y las adolescentes, en particular el papel que desempeñan las familias y los pares en la toma de decisiones (19), la necesaria interfaz entre el sistema educativo y el sistema de salud para prevenir los embarazos no intencionales (20), el involucramiento de los adolescentes varones (21) y el papel de los entornos virtuales en la socialización sexual (22). A ello contribuirá el diseño de intervenciones relacionadas con la educación sexual y la atención en salud sexual y reproductiva que tomen en cuenta los nuevos escenarios, como el que impone la pandemia por COVID-19.

## CONCLUSIONES

La reducción de la tasa de fecundidad en las adolescentes de 15 a 19 años en Uruguay en el último lustro pone fin a 10 años de estancamiento de ese indicador a nivel nacional, si bien no se mantiene esa misma tendencia en menores de 15 años.

Este resultado es producto de muy diversos factores, entre ellos la puesta en marcha de la Estrategia Nacional e Intersectorial de Prevención del Embarazo no Intencional en Adolescentes, la implementación de otras políticas intersectoriales y la existencia de una legislación robusta en materia de derechos humanos e igualdad de género. En este sentido, se deben resaltar las políticas de educación sexual, y de salud sexual y reproductiva, así como el mayor acceso a una amplia canasta de anticonceptivos en el sistema de salud.

Es necesario asegurar la continuidad de las políticas públicas, como la estrategia nacional aprobada, de modo de contar con políticas de Estado ajustadas a un enfoque de derechos humanos y de género transformativo. A más corto plazo, es especialmente relevante analizar el impacto de la pandemia de COVID-19 en el bienestar, el acceso a los derechos sexuales y reproductivos, y la salud integral de la población adolescente.

## Declaración.

Las opiniones expresadas en este artículo son responsabilidad de los autores y no necesariamente reflejan las opiniones, políticas o posiciones oficiales de las instituciones a las cuales están afiliados, ni los criterios ni la política de la *Revista Panamericana de Salud Pública / Pan American Journal of PublicHealth* y/o de la Organización Panamericana de la Salud.
